# Luteolin Modulates 6-Hydroxydopamine-Induced Transcriptional Changes of Stress Response Pathways in PC12 Cells

**DOI:** 10.1371/journal.pone.0097880

**Published:** 2014-05-20

**Authors:** Ling-Wei Hu, Jui-Hung Yen, Yi-Ting Shen, Kuan-Yi Wu, Ming-Jiuan Wu

**Affiliations:** 1 Department of Biotechnology, Chia Nan University of Pharmacy and Science, Tainan, Taiwan; 2 Department of Molecular Biology and Human Genetics, Tzu Chi University, Hualien, Taiwan; 3 Department of Pharmacy, Chia Nan University of Pharmacy and Science, Tainan, Taiwan; Taipei Medical University, Taiwan

## Abstract

The neurotoxin 6-hydroxydopamine (6-OHDA), which causes transcriptional changes associated with oxidative and proteotoxic stress, has been widely used to generate an experimental model of Parkinson’s disease. The food-derived compound luteolin has multi-target actions including antioxidant, anti-inflammatory and neurotrophic activities. The aim of this study is to investigate how luteolin affects 6-OHDA-mediated stress response pathways. The results showed that when PC12 cells were pre-treated with luteolin (20 µM) 30 min prior to 6-OHDA (100 µM) exposure, 6-OHDA-induced ROS overproduction, cytotoxicity, caspase-3 activation, and mRNA expression of BIM, TRB3 and GADD34 were significantly attenuated. Moreover, 6-OHDA-mediated cell cycle arrest and transcription of p53 target genes, p21, GADD45α and PUMA, were reduced by luteolin. Luteolin also significantly down-regulated 6-OHDA-mediated unfolded protein response (UPR), leading to decreases in phospho-eIF2α, ATF4, GRP78 and CHOP. In addition, luteolin attenuated 6-OHDA-induced Nrf2-mediated HO-1 and GCLC. Taken together, these results suggest that diminishing intracellular ROS formation and down-regulation of p53, UPR and Nrf2-ARE pathways may be involved in the neuroprotective effect of luteolin.

## Introduction

Parkinson’s disease (PD) is a progressive neurodegenerative condition characterized by the loss of dopaminergic neurons in the substantia nigra pars compacta and/or the presence of Lewy bodies, which are mainly composed by fibrillary aggregated α-synuclein, within neurons [1]. A growing body of evidence indicates that elevated oxidative stress and the pro-inflammatory response occur early in the development of the disease and these processes contribute to and exacerbate nigrostriatal degeneration [2]. Most insights into the pathogenesis of PD come from investigations performed in experimental animal and cell models, especially those that apply neurotoxins [3]. Two of the most commonly studied models involve the neurotoxins, 1-methyl-4-phenylpyridinium (MPP^+^) and 6-hydroxydopamine (6-OHDA). 6-OHDA, which shares structural similarities with dopamine and norepinephrine, is selectively taken up by catecholaminergic neurons, and causes their damage or death [4]. 6-OHDA destroys catecholaminergic structures by the combined effect of reactive oxygen species (ROS) and quinones. It is thought that the ROS initiate cellular oxidative stress and *p*-quinone mediates 6-OHDA-induced cell death [5].

A large number of tightly regulated stress response pathways have evolved to allow cells to cope with and manage different types of cell stress. Primary among the transcriptional responses to stress are the p53 pathway, endoplasimic reticulum (ER) stress responses, the antioxidant response, and the heat shock response (HSR) [6], [7]. It has been reported that 6-OHDA-generated ROS induce DNA damage and subsequent activation of p53, and the expression of PUMA (p53-upregulated mediator of apoptosis), which is required for cell death in murine midbrain neurons [8].

There is overwhelming evidence that aggregation and accumulation of misfolded proteins is responsible for neurodegeneration. Instrumental in protein quality control in the ER is the unfolded protein response (UPR), which is activated upon ER stress to reestablish homeostasis through a sophisticated transcriptionally and translationally regulated signaling network [9], [10]. In addition to upregulating the genes that support adaptation to and recovery from ER stress, prolonged ER stress induces pro-apoptotic pathways [11]. UPR activation markers, phosphorylated PERK (double-stranded RNA-dependent protein kinase-like endoplasmic reticulum kinase) and phosphorylated eIF2α (eukaryotic initiation factor 2α) are up-regulated in neuromelanin containing dopaminergic neurons in the substantia nigra of PD patients [12]. Studies of familial PD lend further support to the presence of ER stress and subsequent neuron death in the affected cells [13], [14]. Recent research reveals that the activation of the UPR coincides with the initiation of apoptosis in the α-synuclein over-expression model of PD [15]. 6-OHDA-induced protein oxidation also causes upregulation of UPR. It has also been reported that 6-OHDA induces expression of genes associated with UPR in MN9D cells [16], [17], human neuroblastoma SH-SY5Y cells [18] and rat pheochromocytoma PC12 cells [4], [19].

The main signaling pathway involved in the oxidative stress response is the Keap1-Nrf2-ARE pathway. Nrf2 induces expression of antioxidant enzymes, such as heme oxygenase-1 (HO-1), glutamatecysteine ligase (GCL), catalase, glutathione dismutase, and superoxide dismutase (SOD) [20]. Many quinones (such as *o*- and *p*-quinone) are Michael acceptors, which can react with the critical cysteine thiolate (soft base) groups in Keap1 and consequently suppress Nrf2 ubiquitination and induce the expression of ARE-mediated enzymes [21].

Luteolin (3′,4′,5,7-tetrahydroxyflavone, **Fig. S1** in File S1), is a naturally occurring flavonoid and exhibits antioxidant, anti-inflammatory, antimicrobial and anticancer activities [22]. Luteolin has multiple bioactivities and neuroprotective effects, exhibits anti-inflammatory activity in microglia [23] and attenuates neurotoxicities induced by peroxide [24], amyloid β (Aβ) protein [25] and 6-OHDA [26], [27] in cell culture. Luteolin can cross the blood-brain barrier (BBB) and has anti-amnesic effects against the toxicity of Aβ in mice while attenuating scopolamine-induced amnesia in rats [28], [29]. Nevertheless, the molecular mechanism underlying its neuroprotective activity against 6-OHDA-induced cytotoxicity remains unclear. The specific aim of this study is thus to investigate how luteolin affects 6-OHDA-stimulated cellular stress responses, namely, the p53, ER-UPR and Nrf2-ARE pathways, in PC12 cells, and the results may provide valuable insights into the mechanisms underlying its neuroprotective effects.

## Materials and Methods

### Chemicals

6-Hydroxydopamine (6-OHDA), luteolin, MTT (3-[4, 5-dimethylthiazol-2-yl]-2, 5-diphenyl tetrazolium bromide), propidium iodide (PI), RNase, tunicamycin, α-lipoic acid (LA), sodium dihydroxybenzene disulfonate (tiron), and RPMI-1640 medium, as well as other chemicals, were purchased from Sigma-Aldrich Co. (St. Louis, MO), unless otherwise indicated.

### Cell Culture

The rat adrenal pheochromocytoma cell line, PC12, was obtained from the Bioresource Collection and Research Center (Hsinchu, Taiwan). It is a widely studied model of neuronal differentiation and cell death [30]. PC12 cells were maintained in RPMI-1640 medium (Sigma Chemical), which contains 2 mM glutamine, 1.5 g/l sodium bicarbonate, 4.5 g/l glucose, 10 mM HEPES, 1 mM sodium pyruvate, and 100 U/ml penicillin and streptomycin, supplemented with 10% heat-inactivated equine serum (Hyclone, Logan, UT, USA) and 5% fetal bovine serum (Invitrogen, Carlsbad, CA, USA) in a 5% CO_2_ incubator at 37°C.

### Drug Treatments and Cell Viability Assay

PC12 cells (1×10^6^/ml) were seeded in 6-well plates in serum-free RPMI-1640 medium and then pretreated with the indicated reagent or an equivalent volume of DMSO vehicle (final concentration of 0.1%) for 30 min before treatment with 6-OHDA (stock solution 10 mM in ascorbic acid 10 mM) [31]. Cell viability after 6-OHDA insult was assessed by the mitochondrial-dependent reduction of 3-(4, 5-dimethylthiazol-2-yl)-2, 5-diphenyl tetrazolium bromide (MTT) to purple formazan and was quantitated by measurement of A_550_ [32]. Cell viability was then reconfirmed by Calcein AM (Invitrogen) assay. Briefly, cells were incubated with 5 µM Calcein AM for 30 min at 37°C, and the fluorescent signal was monitored using 485 nm excitation and 530 nm emission [33].

### Cell Cycle Analysis

After exposure to 6-OHDA for 8 h, PC12 cells were washed twice with PBS and fixed with ice-cold 70% ethanol overnight. The cells were then centrifuged at 1500×g for 10 min and washed with phosphate-buffered saline (PBS) twice. Cells were stained by the addition of 1 ml DNA-staining solution (20 µg/ml of propidium iodide and 50 µg/ml of RNase) and incubated in the dark at 4°C for 15 min before flow cytometry analysis (FACScan, BD Biosciences, San Jose, CA, USA). To evaluate the cell cycle, PI fluorescence was collected as FL2 (linear scale) using Modfit LT 3.2 (Verity Software House).

### Intraceullular ROS Analysis

Flow cytometry was used to analyze intracellular reactive oxygen species with the fluorescence probe 2′,7′-dichlorodihydrofluorescein diacetate (H_2_DCFDA) (Invitrogen), which passively diffuses into the cell and is cleaved and oxidized to 2′,7′-dichlorofluorescein. PC12 cells (1×10^6^/ml) were seeded in 6-well plates in serum-free RPMI-1640 medium and then treated with test reagent or an equivalent volume of DMSO vehicle (final concentration of 0.1%) for 30 min, and then loaded with 5 µM H_2_DCFDA and 100 µM 6-OHDA for 30 min. Cells were then washed twice with cold PBS and analyzed immediately [34]. Three independent samples of 10,000 cells were analyzed for each experimental condition, and the mean fluorescence intensity (MFI) was obtained.

### RNA Extraction, Reverse Transcription Real-time PCR and Semi-quantitative RT-PCR

PC12 cells (1×10^6^/ml) were seeded on 6-well plates in serum-free RPMI-1640 medium and treated with the indicated reagent or with an equivalent volume of DMSO vehicle (final concentration of 0.1%) for the indicated period [34]. Total cellular RNA was prepared using Illustra RNAspin Mini RNA Isolation Kit (GE Healthcare, Buckinghamshire, UK). Reverse-transcription was carried out using 0.8 µg RNA and High-Capacity cDNA Archive kit (Applied Biosystems, Foster City, CA, USA). Quantitative PCR was performed with 2 µl cDNA obtained, as obtained above, in 25 µl solution containing 200 nM primers **(** **Table 1**) and Power SYBR Green PCR Master Mix (Life Technologies). Amplification was conducted in a StepOne Real-Time PCR Systems (Life Technologies). PCR conditions were as follows: 95°C for 2 min, 40 cycles at 94°C for 15 s, and 60°C for 60 s. Target gene expression was determined using the ΔΔCT method and was normalized to the respective β-actin expression levels. The identity and purity of the amplified product was checked through analysis of the melting curve carried out at the end of amplification process.

**Table 1:** Primer pairs used in RT-Q-PCR.

Gene		Primer Sequence (5′–3′)	Amplicon (bp)
β-actin [80]	Forward	CCTCTGAACCCTAAGGCCAA	90
	Reverse	AGCCTGGATGGCTACGTACA	
ATF4 [81]	Forward	CTTCTCCAGGTGTTCCTCGT	163
	Reverse	TGCTCAGCCCTCTTCTTCTG	
ATF6α	Forward	CGGTCCACAGACTCCTGTTC	95
	Reverse	CTGTCGCCATACAAGGAAAGG	
BIM	Forward	AGAATCGCAAGACAGGAGCCCG	75
	Reverse	CCTGGCAAGGAGGACTTGGGGT	
CHOP [36]	Forward	AAGAATCAAAAACC TTCACTACTCTTGACC	91
	Reverse	TGGGAGGTGCTTGTGACCTCTGC	
GADD34 [36]	Forward	TGCTCGACGCATTGCCCAGG	82
	Reverse	AAGGCGTGTCCATGCTCTGGC	
GADD45α [82]	Forward	TGGCTGCGGATGAAGATGAC	75
	Reverse	CGCAACAGAAAGCACGAATGA	
GCLC [83]	Forward	TGGCCAGCCGTACGGAGGAA	143
	Reverse	CAGGGAGCCTAGCCTGGGA	
GRP78 [36]	Forward	CAACTCACGTCCAACCCGGAGAA	171
	Reverse	TGTCTTGGTTTGCCCACCTCCG	
HO-1 [84]	Forward	GCCTGCTAGCCTGGTTCAAG	87
	Reverse	AGCGGTGTCTGGGATGAACTA	
p21	Forward	ATTCTCGACACAGCAGGTCA	75
	Reverse	AGAAAGCCCTCCCCAGTTCT	
p53	Forward	CTACTTCCCAGCAGGGTGTC	86
	Reverse	GGGAGCTCGATGCTCATATCC	
PUMA [81]	Forward	GAGCCAAACCTGACCACTA	170
	Reverse	GCACAGGATTCACAGTCTG	
TRB3 [81]	Forward	GGACAAGATGCGAGCCACAT	179
	Reverse	CCACAGCAGGTGACAAGTCT	

With regard to the semi-quantative mRNA levels of XBP1s and XBP1u, the cDNA product, obtained above, was also subjected to 30 cycles of PCR using the forward primer TTACGAGAGAAAACTCATGGGC [35] and reverse primer GCGTCAGAATCCATGGGA [36] specific for rat XBP1, followed by 3% agarose gel electrophoresis.

### Protein Extraction, SDS/PAGE and Immunoblotting

PC12 cells (1×10^6^/ml) were seeded on 100 mm dishes in serum-free medium and exposed to the indicated reagent for the indicated period. Cells were washed with PBS, scraped with ice cold RIPA buffer (Thermo Fisher Scientific, Inc., Rockford, IL) and then incubated on ice for 30 min. The cellular debris was removed by centrifugation (8,000×g for 15 min) at 4°C and the cell lysate was carefully transferred to the microcentrifuge tube. The protein concentration was measured by the Bradford method (Bio-Rad Laboratories, Hercules, CA, USA) using bovine serum albumin as a standard.

Cell lysates were separated on 10% SDS-PAGE and transferred onto Hybond-P PVDF (GE Healthcare) at 20 volts overnight at 4°C. The membranes were blocked at 4°C in PBST blocking buffer (5% BSA in PBS with 0.05% Tween 20, pH 7.4) for 8 h. Blots were analyzed with each antibody (**Table 2**) at a dilution of 1∶1000–1∶5000 overnight at 4°C. After three washes with PBST, the blots were incubated with suitable horseradish peroxidase-conjugated secondary antibody (Jackson ImmunoResearch, West Grove, PA) at a dilution of 1∶10,000 for 1 h. The blots were washed again and the proteins of interest detected by Amersham ECL Prime Western Blotting Detection Reagents (GE Healthcare), according to the manufacturer’s instructions, and then the chemiluminescence signal was visualized with Hyperfilm ECL X-ray film.

**Table 2:** Primary antibodies used in Western blotting.

Antibody	company	Catalog Number
α-tubulin	Sigma	T 6199
Cleaved caspase-3	BioVision	3015–100
eIF2α	Gene Tex	GTX112919
p-eIF2α	Gene Tex	GTX61039
ATF4	Gene Tex	GTX101943
ATF6α	Santa Cruz	Sc-22799
BiP/GRP78	BD	610978

### SiRNA Knockdown of Nrf2

Scrambled Stealth RNAi negative control duplexes and rat-specific Nrf2 small interfering RNA (siRNA) duplex [5′-CCAUUCCCGAGUUACAGUGUCUUAA-3′ (forward) and 5′-UUAAGACACUGUAACUCGGGAAUGG-3′ (reverse)] were supplied by Invitrogen. PC12 cells were transiently transfected with either Nrf2 siRNA or negative control using Lipofectamine 2000 (Invitrogen) for 18 h, according to the manufacturer’s instructions. The cells were then changed to fresh serum-free medium containing 100 µM 6-OHDA. Knockdown of Nrf2 expression in the cells was confirmed by RT-Q-PCR, and the expression of HO-1 and GCLC were analyzed as described above.

### Statistical Analysis

Experiments were repeated at least three to four times with consistent results. Student’s *t* test was used for comparison between two groups. One-way ANOVA with post-hoc Tukey test was used for comparison between multiple groups. Significance was set at *p*<0.05.

## Results

### Luteolin Reduces 6-OHDA-induced ROS and Restores Cell Viability

**Figure 1:**
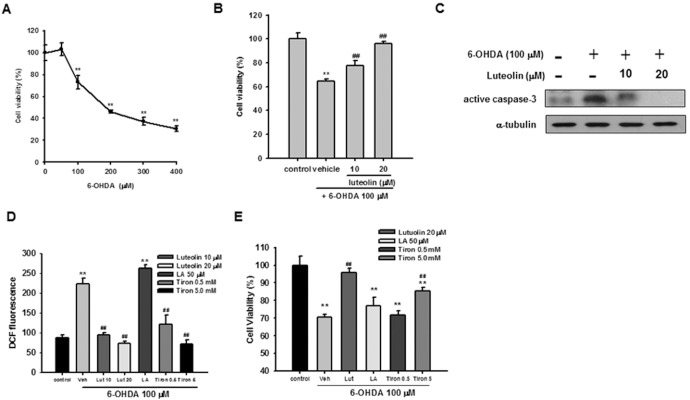
Effects of luteolin on 6-OHDA-induced PC12 apoptosis and ROS over-production. (**A**) PC12 cells (1×10^6^ cells/ml) were treated with different concentrations of 6-OHDA in serum-free medium for 16 h at 37°C. Cell viability was measured by MTT as described in Materials and Methods. (**B**) PC12 cells were treated with luteolin in serum-free medium for 30 min before 6-OHDA (100 µM) insult for 16 h. Cell viability was measured by MTT, as described in Materials and Methods. (**C**) PC12 cells were treated with luteolin in serum-free medium for 30 min before 6-OHDA (100 µM) insult for 12 h. Cell lysates were prepared and immunoblotting was then carried out using an anti-cleaved caspase-3 antibody or anti-α-tubulin antibody. The blots are representative ones from one of three independent experiments. (**D**) PC12 cells (1×10^6^ cells/ml) were treated with antioxidant for 30 min prior to the addition of 6-OHDA for 30 min at 37°C, and ROS production was measured by H_2_DCFDA, as described in the Materials and Methods. (**E**) PC12 cells were treated with antioxidant for 30 min prior to the addition of 6-OHDA for 16 h at 37°C. Cell viability was determined by Calcein-AM cell viability assay. Data represent the mean ± SD of three independent experiments. *, *p*<0.05; **, *p*<0.01 represent significant differences compared with vehicle control (without 6-OHDA).

Increasing evidence suggests that PD mimetic 6-OHDA exposure causes PC12 cell death through oxidative stress [17]. **Figure 1A** shows that PC12 cells treated with 6-OHDA (0–400 µM) for 16 h exhibited a dose-dependent cytotoxicity, as measured by MTT assay and reconfirmed by Calcein AM staining as described in the Materials and Methods section. 6-OHDA caused about 30% and 70% cell death at concentrations of 100 µM and 400 µM, respectively. In addition, the 6-OHDA-mediated cytotoxicity in PC12 cells was time-dependent (**Fig. S2** in File S1).

PC12 cells were treated with luteolin (10 and 20 µM) 30 min before exposure to 100 µM 6-OHDA for 16 h. Cell viabilities were significantly elevated by luteolin in a dose-dependent manner (*p*<0.01), and 20 µM luteolin almost completely ameliorated the cytotoxicity induced by 6-OHDA (**Fig. 1B**).

Caspase-3 is considered to be the most important of the executioner caspases, and ultimately causes the morphological and biochemical changes seen in apoptotic cells [37]. We found that treatment of PC12 cells with 6-OHDA (100 µM) for 12 h caused caspase-3 activation. Co-treatment of cells with luteolin (10 and 20 µM) and 6-OHDA (100 µM) dose-dependently attenuated active caspase-3 levels (**Fig. 1C**). These results indicate that luteolin inhibits 6-OHDA-mediated PC12 apoptosis.

It has been postulated that H_2_O_2_-dependent and -independent actions are involved in 6-OHDA-induced PC12 cell death [38]. Added 6-OHDA is rapidly oxidized and generates both H_2_O_2_ and *p*-quinone, and both agents can induce caspase-3 activation [5]. We therefore studied whether luteolin could attenuate 6-OHDA-stimulated PC12 cell death through reducing intracellular ROS production. **Figure 1D** demonstrates that exposure of PC12 cells to 100 µM 6-OHDA for 30 min increased DCF fluorescence. Pre-incubation of PC12 cells with 10 and 20 µM luteolin 30 min prior to 6-OHDA insult prevented the intracellular oxidation of the fluorescent probe.

Previous research has demonstrated that the addition of catalase, an H_2_O_2_-removing enzyme, completely abolished the cytotoxic effect of H_2_O_2_, while a significant but partial protective effect was observed against that of 6-OHDA [5]. In the current study we examined the effects of two other ROS scavengers, tiron and α-lipoic acid. Tiron (sodium dihydroxybenzene disulfonate) is a cellular permeable superoxide scavenger, and high concentration of tiron has been shown to partially prevent 6-OHDA-induced PC12 cell death [39]. α-lipoic acid (1,2-dithiolane-3-pentanoic acid; LA) is a direct scavenger of ROS/RNS *in* *vitro*, and an effector of important cellular stress response pathways that ultimately influence endogenous cellular antioxidant levels and reduce proinflammatory mechanisms [40], while also serving as a potential alternative therapy for PD [41]. We found that the thiol reductive agent LA (50 µM) did not change intracellular ROS level or protect PC12 cells from 6-OHDA-induced cytotoxicity (**Fig. 1D and 1E**). On the other hand, tiron (0.5 mM and 5 mM) inhibited 6-OHDA-mediated ROS production in PC12 cells in a dose-dependent manner, but only 5 mM tiron exhibited a cytoprotective effect. The fact that complete depletion of ROS by tiron (5 mM) only partially restored cell viability supports the earlier notion that, in addition to oxidative stress, 6-OHDA-induced cell death may result from other pathways [5]. Furthermore, the higher efficacy of luteolin may be attributed not only to its direct ROS scavenging activity, but also to modulating other signaling pathways [42], [43].

### Luteolin Relieves 6-OHDA-provoked-cell Cycle Arrest and Inhibits Transcriptional Activation of the p53 Pathway

**Figure 2:**
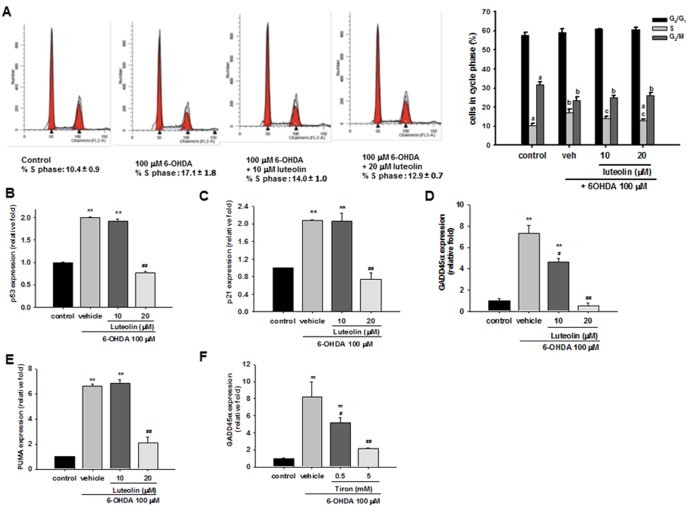
Effects of luteolin on 6-OHDA-mediated cell cycle arrest and gene expression of the p53 pathway in PC12 cells. (**A**) PC12 cells were treated with 6-OHDA for 8 h and then fixated with ethanol overnight, and stained with PI/RNase. Cell cycle was analyzed by flow cytometry, as described in the Materials and Methods. Numbers indicate the percentage of cells in G_0_/G_1_, S and G_2_/M phases from three separate analyses. Different letters denote statistically significant differences in mean (*p*<0.05). (**B–F**) PC12 cells were treated with luteolin (10 or 20 µM) or tiron (0.5 or 5 mM) for 30 min before 6-OHDA (100 µM) insult for 8 h. RNA was then prepared for RT-Q-PCR analysis of p53, p21, GADD45α and PUMA. Data represent the mean ± SD of three independent experiments. *, *p*<0.05; **, *p*<0.01 represent significant differences compared with the vehicle control (without 6-OHDA). #, *p*<0.05; ##, *p*<0.01 represent significant differences compared with 6-OHDA-treated vehicle.

We further investigated how 6-OHDA affects the cell population distribution in the cell cycle by staining the cellular DNA with propidium iodide (PI). Studies of cell cycle progression following partial synchronization by culturing in serum-free medium show that 100 µM 6-OHDA exposure for 8 h caused an obvious emergence of the S phase (17.06%±1.84%) compared with the control (10.37%±0.96%), as well as a decrease in the number of cells in the G_2_/M phase (**Fig. 2A**). Because cell proliferation is inhibited by 6-OHDA (**Figs. 1A and B**), it seems unlikely that the increase in the S phase fraction represents an increase in cells that are actively replicating DNA, but more likely this indicates an accumulation of cells whose progress is arrested in this phase of the cell cycle [44]. Addition of 10 and 20 µM luteolin resulted in a significantly decreased percentage of cells in the S phase without increasing that in the G_2_/M phase. In accordance with the cell viability results, these data indicated that luteolin decreased 6-OHDA-induced S phase arrest in PC12 cells.

The p53 pathway is turned on in response to multiple upstream events, and thus can be activated by xenobiotics with genotoxic or oxidative activity [7]. We found that p53 mRNA expression was induced by 6-OHDA (100 µM) in a time-dependent manner (**Fig. S3** in File S1). Moreover, addition of 20 µM luteolin could significantly decrease p53 over-expression (**Fig. 2B**).

An increase in p53 leads to the induction of growth arrest genes such as p21, which is a universal cyclin dependent kinase inhibitor implicated in cell cycle arrest, and the growth arrest and DNA damage inducible gene 45 (GADD45), which functions as a stress sensor [45]. In parallel to p53 expression, the stimulation of p21 mRNA by 6-OHDA (100 µM) increased with time (from 8 to 12 h incubation) (**Fig. S3** in File S1). Treatment of PC12 cells with a higher concentration of luteolin (20 µM) completely blocked p21 induction (**Fig. 2C**). 6-OHDA (100 µM) induced increased GADD45α mRNA expression by 7.4- and 10.6-fold after incubation for 8 and 12 h, respectively (**Fig. S3** in File S1). Treatment of PC12 cells with luteolin dose-dependently attenuated 6-OHDA-mediated GADD45α upregulation (**Fig. 2D**), and 20 µM luteolin completely blocked the induction.

It has been reported that the mRNA expression of PUMA, the p53 target gene, is stimulated by 6-OHDA [4], [19], [46], and its expression is required for death caused by 6-OHDA in differentiated PC12 cells [46]. We found that PUMA was increased by 6.4- and 5.2-fold after exposure to 6-OHDA 8 and 12 h, respectively (**Fig. S3** in File S1). Treatment of PC12 cells with 20 µM luteolin for 30 min before exposure to 6-OHDA (100 µM) for 8 h significantly attenuated PUMA upregulation (**Fig. 2E**).

We further investigated whether 6-OHDA-mediated ROS overproduction serves as a direct trigger of the p53 pathway. We found that p53-responsive GADD45α was significantly attenuated by tiron in a dose dependent manner (**Fig. 2F**), indicating the involvement of ROS in the transcriptional activation of p53 pathway and that the inhibitory effect of luteolin (20 µM) operates at least in part through attenuation of ROS production.

### Luteolin Inhibits 6-OHDA-mediated eIF2α-ATF4 Activation and ATF4 Transcription in PC12 Cells

Microarray analysis of RNA collected from 6-OHDA-treated MN9D cells showed that ER stress-related genes, ATF3, ATF4, and CHOP (C/EBP homologous protein/Gadd153), were upregulated [17]. Exposure of differentiated PC12 cells to 100 µM 6-OHDA for 8 h also induced expression of mRNA coding ER stress and UPR, such as GRP78/Bip, ATF4, CHOP, and GADD34 [4]. Furthermore, it was found that ER stress induced by both H_2_O_2_ and quinone might be responsible for the neurotoxicity of 6-OHDA [38]. We therefore also examined how luteolin affects 6-OHDA-induced unfolded protein response (UPR) in PC12 cells.

**Figure 3:**
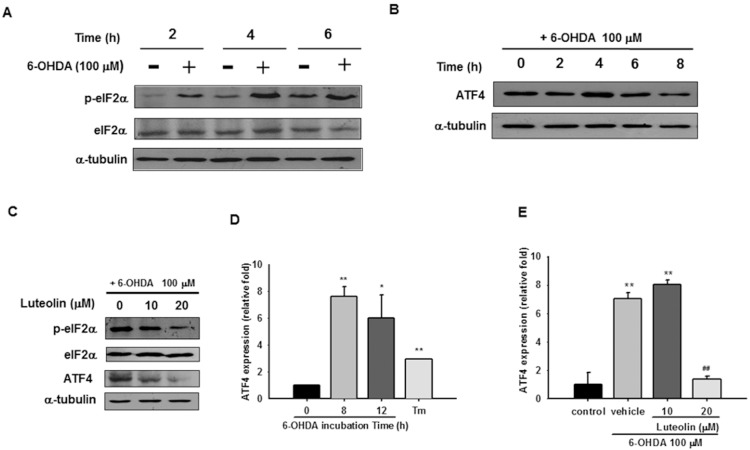
Effects of luteolin on 6-OHDA-mediated eIF2α-ATF4 activation. (**A**) PC12 cells (1×10^6^ cells/ml) were cultured in serum-free medium and then incubated with or without 6-OHDA (100 µM) for 2, 4, or 6 h. Cell lysates were prepared and immunoblotting was then carried out with antibodies against anti-p-eIF2α, anti-eIF2α and anti-α-tubulin. (**B**) PC12 cells were incubated with 6-OHDA (100 µM) for 0, 2, 4, 6, or 8 h. Cell lysates were prepared and immunoblotting was then carried out with antibodies against anti-ATF4 and anti-α-tubulin. (**C**) Cell lysates prepared from those co-treated with 6-OHDA (100 µM) and indicated concentration of luteolin for 4 h were subjected to p-eIF2α, eIF2α, ATF4, and α-tubulin analysis as described in Materials and Methods_._ These blots are representative ones from one of three independent experiments. (**D**) Changes in ATF4 mRNA expression after being incubated with 6-OHDA (100 µM) for 8 and 12 h. Cells treated with tunicamycin (Tm, 1 µg/ml) for 8 h served as a positive control. ATF4 mRNA expression was measured by RT-Q-PCR and normalized to β-actin, as described in the Materials and Methods. (**E**) Effect of luteolin on ATF4 mRNA expression. PC12 cells were treated with luteolin (10 or 20 µM) for 30 min before 6-OHDA (100 µM) insult for 8 h. RNA was then prepared for RT-Q-PCR analysis. The data represent the mean ± SD of three independent experiments. *, *p*<0.05; **, *p*<0.01 represent significant differences compared with the vehicle control (without 6-OHDA). ##, *p*<0.01 represents significant differences compared with the 6-OHDA-treated vehicle.

Transient global translation is initially stalled during ER stress by the PERK signaling pathway [47], which allows PERK to phosphorylate and inactivate the translation initiation factor eIF2α. Phosphorylated eIF2α selectively enhances translation of ATF4 [48], [49]. We first examined how 6-OHDA affects the eIF2α-ATF4 pathway. PC12 cells were incubated with 100 µM 6-OHDA for various periods, and total cell lysates were prepared and subjected to Western blot analysis. **Figure 3A** shows that treatment with 100 µM 6-OHDA for 2 to 4 h led to an increase in eIF2α phosphorylation, and then decreased after 6 h. Furthermore, treatment of PC12 cells with 100 µM 6-OHDA for 4 h led to a transient increase in the level of ATF4 protein (**Fig. 3B**). Treatment of PC12 cells with luteolin (10 and 20 µM) 30 min before 6-OHDA (100 µM) insult significantly inhibited eIF2α phosphorylation and decreased the level of ATF4 protein (**Fig. 3C**).

Not only does 6-OHDA exposure induce translation of pre-existing ATF4 mRNA, it has also been related to an increase in ATF4 mRNA expression [4], [50]. **Figure 3D** shows that PC12 cells exposed to 100 µM 6-OHDA for 8–12 h induced an increase in ATF4 mRNA expression by 6- to 8-fold as compared with the vehicle control. ATF4 was also significantly upregulated by 3-fold by the UPR inducer tunicamycin (1 µg/ml). These results indicate that ATF4 expression was induced transcriptionally and translationally by 6-OHDA. Co-treated with 20 µM luteolin significantly lowered 6-OHDA-mediated ATF4 mRNA expression (**Fig. 3E**).

### 6-OHDA Induces ATF6α mRNA Expression, but does not Activate Processing of ATF6α or XBP1

**Figure 4:**
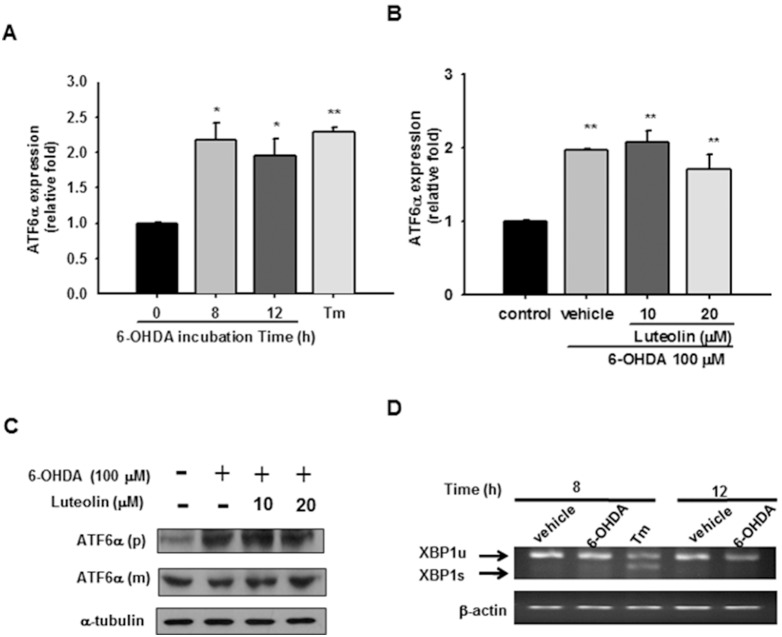
Effects of 6-OHDA on ATF6α transcription and processing as well as XBP1 splicing. (**A**) Changes in ATF6α mRNA expression after being incubated with 6-OHDA (100 µM) for 8 and 12 h. Cells treated with tunicamycin (Tm, 1 µg/ml) for 8 h served as a positive control. ATF6α mRNA expression was measured by RT-Q-PCR and normalized to β-actin, as described in the Materials and Methods. (**B**) Effect of luteolin on ATF6α mRNA expression. PC12 cells were treated with luteolin (10 or 20 µM) for 30 min before 6-OHDA (100 µM) insult for 8 h. RNA was then prepared for RT-Q-PCR analysis. The data represent the mean ± SD of three independent experiments. *, *p*<0.05; **, *p*<0.01 represent significant differences compared with vehicle control (without 6-OHDA). (**C**) Effect of luteolin on ATF6α protein expression and processing. Cell lysates prepared from those co-treated with 6-OHDA (100 µM) and indicated concentration of luteolin for 12 h were subjected to precursor (p) and mature (m) forms of ATF6α and α-tubulin analysis as described in Materials and Methods_._ These blots are representative from one of three independent experiments. (**D**) Effect of 6-OHDA on XBP1 mRNA splicing in PC12 cells. RNA was prepared from those treated with 6-OHDA (100 µM) for 8 and 12 h and unspliced (u) and spliced (s) forms of XBP1 were measured using PCR followed by agarose gel electrophoresis as described in the Materials and Methods.

It has been reported that ATF6β transcription is upregulated by 6-OHDA in differentiated PC12 cells [4]. However, ATF6α, but not ATF6β, is solely responsible for transcriptional induction of ER chaperones [51]. We thus investigated how luteolin affects 6-OHDA-mediated ATF6α transcription. **Figure 4A** shows that both 6-OHDA (100 µM) and tunicamycin (1 µg/ml) induced about 2-fold increase in ATF6α mRNA as compared with the control after 8 h treatment. Treatment of PC12 cells with luteolin (10 and 20 µM) for 30 min before 6-OHDA (100 µM) insult for 8 h did not change ATF6α mRNA over-expression (**Fig. 4B**).

Upon ER stress, ATF6 is released from the ER chaperone GRP78/Bip, and travels to the Golgi, where it is cleaved by two proteases to release a 50-kDa transcription factor. We thus further investigated whether luteolin inhibits ATF6 activation by Western blotting. **Figure 4C** shows that treatment of PC12 cells with 100 µM 6-OHDA for 12 h caused an increase in the level of full-length ATF6α (p) expression without significantly affecting truncated ATF6α (m) production as compared with the control. This indicates that 6-OHDA had little effect on the activation of ATF6α. Treatment of PC12 cells with luteolin (10 and 20 µM) for 30 min before 6-OHDA (100 µM) insult for 12 h did not alter either the full length or truncated ATF6α levels. All of the above data indicate that the cytoprotective effect of luteolin against 6-OHDA is not involved in modulation of the ATF6 UPR signaling pathway.

ER stress induces IRE1α kinase autophosphorylation, activating the RNase to splice XBP1 mRNA and produce the transcription factor XBP1s [52]. It has been reported that levels of both unprocessed and processed XBP1 mRNA are increased in response to 6-OHDA in MN9D dopaminergic cells [16]. We found that treatment of PC12 cells with the positive control tunicamycin (1 µg/ml) for 8 h significantly enhanced XBP1 processing. On the other hand, there was no significant increase in the processed/active XBP1s after treatment with 6-OHDA for 8 or 12 h in PC12 cells (**Fig. 4D**). This result indicates that 6-OHDA does not significantly activate XBP1 splicing.

### Luteolin Decreases GRP78 and CHOP Expression

**Figure 5:**
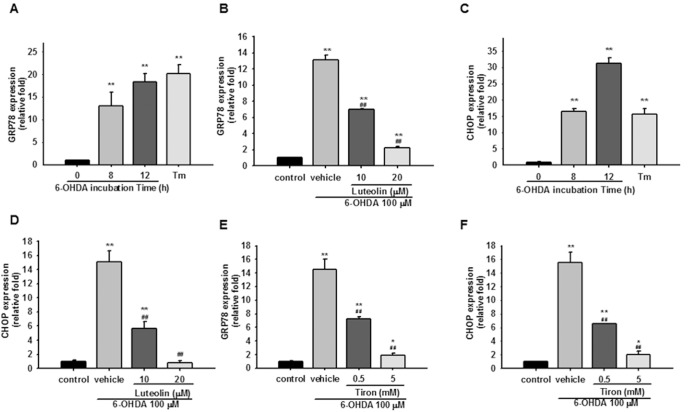
Effects of luteolin on 6-OHDA-mediated GRP78 and CHOP expression in PC12 cells. (**A** **and** **C**) PC12 cells were cultured in serum-free medium and then incubated with 6-OHDA (100 µM) for 8 and 12 h. Cells treated with tunicamycin (Tm, 1 µg/ml) for 8 h served as a positive control. Levels of GRP78 and CHOP mRNA were measured by RT-Q-PCR and normalized to β-actin as described in the Materials and Methods. (**B, D, E and F**) PC12 cells were treated with luteolin or tiron for 30 min before 6-OHDA (100 µM) insult for 8 h. RNA was then prepared for RT-Q-PCR analysis. Data represent the mean ± SD of three independent experiments. **, *p*<0.01 represents significant differences compared with the vehicle control (without 6-OHDA). ##, *p*<0.01 represents significant differences compared with the 6-OHDA-treated vehicle.

The major cellular targets of the UPR are GRP78/BiP and CHOP/GADD153 and their induction is regulated primarily at the transcriptional level. GRP78/Bip, an ER chaperone with anti-apoptotic properties, not only binds to unfolded proteins, but also regulates the activation of ER stress signal transducers. **Figure 5A** shows that 100 µM 6-OHDA stimulated increases in GRP78 mRNA expression by about 13- and 18-fold after 8 and 12 h treatment, respectively. In comparison, the positive control, tunicamycin (1 µg/ml), induced GRP78 transcription to increase by about 20-fold after 8 h. Treatment of PC12 cells with luteolin (10 and 20 µM) for 30 min before exposure to 6-OHDA for 8 h dose-dependently attenuated GRP78 upregulation (**Fig. 5B**).

The results of Western blotting analysis indicated that the most significant increase in GRP78 protein was observed after exposure to 100 µM 6-OHDA for 12 h. Addition of luteolin (10 and 20 µM) decreased the GRP78 protein level in a dose-dependent manner (**Fig. S4** in File S1).

Expression of CHOP is crucial for the development of programmed cell death and regeneration. Our results show that its expression was increased by about 16- and 31-fold after 6-OHDA (100 µM) treatment for 8 and 12 h, respectively. The positive control, tunicamycin (1 µg/ml), also led to a ∼16-fold increase in CHOP expression after 8 h incubation (**Fig. 5C**). Treatment of PC12 cells with luteolin dose-dependently attenuated 6-OHDA-mediated CHOP upregulation, which was completely inhibited by 20 µM luteolin (**Fig. 5D**).

It has been reported that both H_2_O_2_ and *p*-quinone play a significant role in the induction of CHOP via ER stress response in PC12 cells [38]. We further investigated whether 6-OHDA-mediated ROS overproduction is involved in inducing the gene expression involved in UPR. We found that 6-OHDA-upregulated transcription of ER stress markers, GRP78 and CHOP, were significantly ameliorated by tiron (05 and 5 mM) in a dose dependent manner (**Fig. 5E and F**). These data suggest that in addition to triggering the adaptive phase of the UPR, 6-OHDA also activates the apoptotic UPR response, and that 6-OHDA-mediated UPR is ROS-dependent in PC12 cells. Luteolin (20 µM) could completely block 6-OHDA-mediated ROS overproduction and CHOP expression, but only partially inhibited GRP78 upregulation. Because UPR mediated cell survival or death is regulated by the balance of GRP78 and CHOP expression, the preferential inhibition of CHOP over GRP78 by luteolin may reflect its cytoprotective activity.

### Luteolin Modulates Nrf2 Target Gene Expression

**Figure 6:**
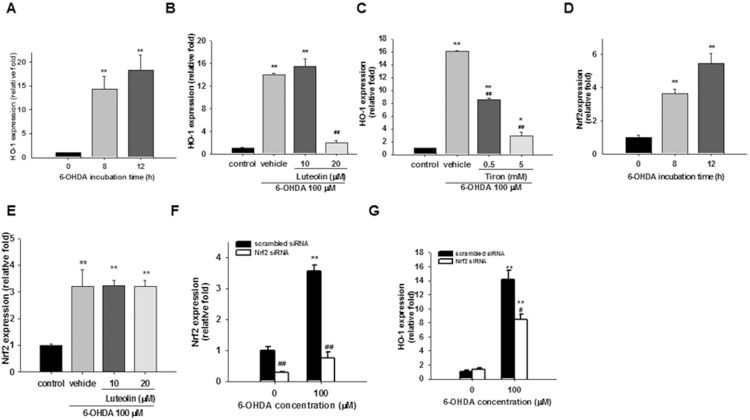
Effects of luteolin on Nrf2 target gene expression. (**A and D**) PC12 cells were cultured in serum-free medium and then incubated with 6-OHDA (100 µM) for 8 and 12 h. Levels of HO-1 and Nrf2 mRNA were measured by RT-Q-PCR and normalized to β-actin as described in the Materials and Methods. (**B, C, and E**) PC12 cells were treated with luteolin (10 or 20 µM) or tiron (0.5 and 5 mM) for 30 min before 6-OHDA (100 µM) insult for 8 h. RNA was then prepared for RT-Q-PCR analysis. Data represent the mean ± SD of three independent experiments. *, *p*<0.05; **, *p*<0.01 represent significant differences compared with the vehicle control (without 6-OHDA). ##, *p*<0.01 represents significant differences compared with the 6-OHDA-treated vehicle. (**F and G**) Contribution of Nrf2 activation to HO-1 expression in response to 6-OHDA. PC12 cells were transfected transiently with nonspecific (scrambled) siRNA or with Nrf2-specific siRNA before 6-OHDA (100 µM) treatment. Effects of Nrf2 siRNA on the expression of Nrf2 and HO-1 after 6-OHDA treatment for 8 h were measured by RT-Q-PCR. Data represent the mean ± SD of three independent experiments. **p<0.01 represents significant differences compared with the vehicle control (without 6-OHDA). #, p<0.05; ##, *p*<0.01 represent significant differences compared with the scrambled siRNA-transfected group.

Upon ER stress, PERK phosphorylates Nrf2, resulting in dissociation of the Nrf2-Keap1 complex, nuclear localization of Nrf2 and activation of transcription by Nrf2 through the antioxidant response element (ARE) [53]–[55]. Nrf2 activation induces expression of antioxidant enzymes, such as heme oxygenase-1 (HO-1) and glutamatecysteine ligase (GCL) [56]. HO-1, an enzyme that degrades heme to biliverdin, free iron and carbon monoxide, is a well-known oxidative stress response protein. In PD, HO-1 is markedly over-expressed in astrocytes of the substatia nigra and Lewy bodies in affected neurons [57]. A dramatic increase in HO-1 transcript was found in response to 6-OHDA in differentiated PC12 cells [4] and in MN9D cells [17]. In this study we found that HO-1 mRNA expression was dramatically increased after 6-OHDA treatment for 8 and 12 h in PC12 cells (**Fig. 6A**). Previously, we found that luteolin alone stimulated HO-1 expression, which contributes, at least in part, to the cytoprotective effect of luteolin in PC12 cells in serum-free medium [42]. In the current work we found that, in combination with 6-OHDA, luteolin (20 µM) otherwise inhibited HO-1 expression (**Fig. 6B** **)**. Western blotting also showed that HO-1 protein expression was elevated after exposure to 6-OHDA for 8 h, and remained high after 12 h. Addition of luteolin (20 µM) also significantly decreased the HO-1 protein level (**Fig. S4** in File S1). Furthermore, the expression of HO-1 was also inhibited by tiron (**Fig. 6C**), indicating the key role of ROS in HO-1 upregulation.

GCL, the GSH synthetic enzyme, is a heterodimeric holoenzyme complex consisting of a catalytic subunit (GCLC), and a modifier subunit (GCLM) [58]. Our results revealed that treatment of PC12 cells with 6-OHDA (100 µM) for 8 and 12 h significantly increased GCLC mRNA expression (**Fig. S5** in File S1). Addition of luteolin (20 M) exhibited a weaker but significant inhibitory effect on GCLC expression (**Fig. S5** in File S1).

Nrf2 expression is subjected to transcriptional, translational, and post-translational regulation [59]. We found that 100 µM 6-OHDA stimulated 3.7- and 5.5-fold Nrf2 mRNA expression after 8 and 12 h treatment, respectively (**Fig. 6D**). However, addition of luteolin (10–20 µM) did not inhibit 6-OHDA-mediated Nrf2 mRNA expression (**Fig. 6E**). To investigate whether Nrf2 indeed serves as an upstream regulator for 6-OHDA-induced HO-1 and GCLC expression, we used Nrf2 siRNA to knock down the expression of Nrf2 in PC12 cells. Nrf2 siRNA-transfected cells were then treated with 6-OHDA (100 µM) for 8 h in serum-free medium, and the expression of Nrf2, HO-1 and GCLC were determined. **Figure 6F** shows that in scrambled RNA transfected cells, 100 µM 6-OHDA induced about 3.5-fold increase in Nrf2 mRNA expression as compared with the control, and Nrf2 siRNA successfully knocked down ∼80% of endogenous and 6-OHDA-induced Nrf2 expression. **Figure 6G** shows that when PC12 cells were transfected with scrambled RNA and then treated with 6-OHDA for 8 h, the expression of HO-1 increased significantly (*p*<0.01). In Nrf2 siRNA-transfected cells, a reduction of about 40% was found for 6-OHDA-induced, but not endogenous, HO-1 expression as compared with those of scrambled siRNA (*p*<0.05). Similar results were also found for 6-OHDA-mediated GCLC expression (data not shown). This confirms that 6-OHDA-mediated HO-1 and GCLC expression is regulated by Nrf2. Luteolin treatment may attenuate Nrf2 activity rather than its expression, and thus down-regulate HO-1 and GCLC expression.

### Luteolin Attenuates 6-OHDA-upregulated Pro-apoptotic Gene Expression

**Figure 7:**
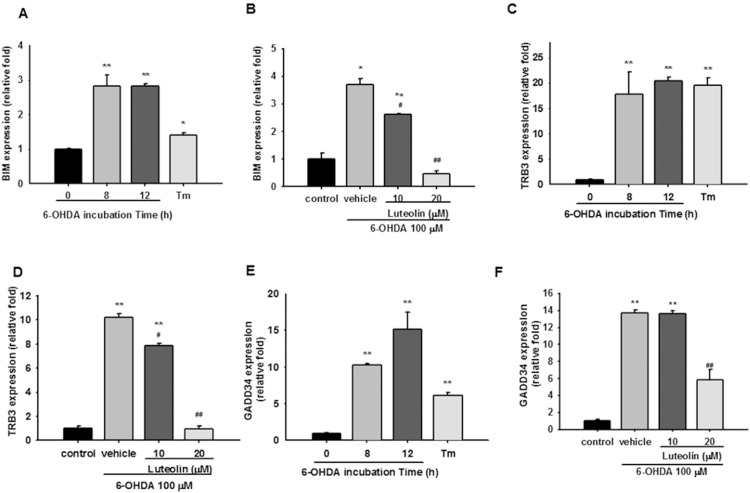
Effects of luteolin on 6-OHDA-mediated pro-apoptotic gene expression in PC12 cells. (**A, C and E**) PC12 cells were cultured in serum-free medium and then incubated with 6-OHDA (100 µM) for 8 and 12 h. Cells treated with tunicamycin (Tm, 1 µg/ml) for 8 h served as a positive control. Levels of BIM, TRB3 and GADD34 mRNA were measured by RT-Q-PCR and normalized to β-actin as described in the Materials and Methods. (**B, D and F**) PC12 cells were treated with luteolin for 30 min before 6-OHDA (100 µM) insult for 8 h. RNA was then prepared for RT-Q-PCR analysis as described in the Materials and Methods. Data represent the mean ± SD of three independent experiments. *, *p*<0.05; **, *p*<0.01 represent significant differences compared with the vehicle control (without 6-OHDA). ##, *p*<0.01 represents significant differences compared with the 6-OHDA-treated vehicle.

It has been reported that neuron death evoked by 6-OHDA is dependent on transcription of genes associated with apoptosis [4]. Several reports addressed the possible role of BH3-only proteins in the modulation of apoptosis under chronic ER stress and activating the mitochondrial component of cell death, and BIM (Bcl-2 interacting mediator of cell death) is one of the most studied of these [60]. **Figure 7A** shows that BIM transcript was induced moderately by 6-OHDA after 8 and 12 h by about three-fold as compared with control (*p*<0.01), while only a 1.4-fold was induced by tunicamycin (1 µg/ml). Treatment of PC12 cells with luteolin dose-dependently attenuated 6-OHDA-mediated BIM over-expression, and 20 µM luteolin completely blocked BIM upregulation (**Fig. 7B**).

TRB3 (tribbles-related protein 3) is a target of CHOP/ATF4, and seems to be involved in CHOP-dependent cell death during ER stress [61]. **Figure 7C** demonstrates that TRB3 transcripts were markedly stimulated by 6-OHDA and tunicamycin. Luteolin (10 and 20 µM) inhibited TRB3 expression in a dose-dependent manner, and 20 µM luteolin completely blocked TRB3 upregulation (**Fig. 7D**). These data are consistent with the results for CHOP, and the expression of these two apoptotic genes was completely inhibited by 20 µM luteolin.

GADD34 is upregulated by ATF4 and encodes a regulatory subunit of an eIF2α-directed phosphatase complex that promotes recovery of protein synthesis in cells exposed to ER stress [62]. We found that 6-OHDA (100 µM) induced more GADD34 expression than tunicamycin (1 µg/ml) did in PC12 cells (**Fig. 7E**). In addition, only 20 µM luteolin could partially inhibit GADD34 expression (**Fig. 7F**).

## Discussion

6-Hydroxydopamine (6-OHDA) is a selective catecholaminergic neurotoxin that has been widely used to generate Parkinson’s disease (PD) models *in* *vitro* and *in* *vivo*. Evidence indicates that extracellular auto-oxidation, which occurs through the generation of toxic products, such as hydrogen peroxide, oxygen-derived radicals, semiquinones, and quinones, plays an important role in 6-OHDA-induced cytotoxicity [5]. The consumption of flavonoid-rich foods and beverages has been suggested to limit the neurodegeneration associated with a variety of neurological disorders, and to prevent or reverse normal or abnormal deteriorations in cognitive performance [63]. Luteolin, a flavone ubiquitously distributed in several types of vegetables, fruits, and medicinal herbs, has antioxidant activity by directly scavenging ROS. Luteolin also inhibits 6-OHDA-induced apoptosis [27] and depresses the 6-OHDA-enhanced Bax/Bcl-2 ratio and p53 expression in PC12 cells [26]. In addition to cytoprotective effects, we have reported that luteolin is a neurotrophic agent [42], and its action is in part through up-regulation of miR-132, thereby activating the cAMP/PKA- and ERK-dependent CREB signaling pathways in PC12 cells [43]. However, little information is available about how luteolin affects transcriptional change of cellular stress response pathways in response to 6-OHDA in PC12 cells.

The results first confirmed that 6-OHDA induced ROS overproduction, caspase-3 activation and cell death. Three different types of antioxidants, namely luteolin, tiron, and lipoic acid (LA), were then used to test their cytoprotective potencies. It has been shown that luteolin can directly quench all kinds of ROS, including superoxide, hydrogen peroxide, singlet oxygen and hydroxyl radical *in* *vitro* [64], [65]. Luteolin also regulates a variety of cell signaling pathways leading to its high neuroprotective efficacy [23], [42], [43]. In addition to being a cellular permeable superoxide scavenger, tiron inhibits the phosphorylation of ROS-induced JNK, which plays a key role in 6-OHDA-induced cell death in PC12 cells [39]. LA acts against free radicals, increases or maintains cellular GSH levels, regulates the redox state in the cells, and affects gene expression [41]. Both luteolin and tiron can block 6-OHDA-mediated ROS production, as detected by reduced DCF fluorescence, and thus significantly restore cell viability. On the other hand, 50 µM LA did not change 6-OHDA-mediated ROS over-production or cell viability. All of these results indicate that ROS is important in mediating the cytotoxicity of 6-OHDA. Luteolin has the catechol moiety, which can be oxidized during antioxidant reaction yielding *o*-quinone and may thus interfere with the cell signaling caused by *p*-quinone, and so exhibit higher cytoprotective efficacy than tiron.

We further found that 6-OHDA treatment for 8 h successfully blocked the progression of cells from the S phase into the G_2_/M phase. In addition to formation of ROS, quinones are Michael acceptors, and cellular damage can occur through alkylation of crucial cellular proteins and DNA [66]. The p53 tumor suppressor induces the transcription of genes that negatively regulate progression of the cell cycle in response to DNA damage [67]. We found that 6-OHDA induced expression of p53 target genes, p21, GADD45α and PUMA, and the interaction with and dissociation of cyclin complexes may result in the cell cycle arrest that was observed in PC12 cells. This result supports an earlier report that 6-OHDA-induced DNA damage leads to the activation of the p53 DNA damage repair pathway, and p53-mediated PUMA upregulation leads to the induction of apoptosis [8]. Pretreatment with luteolin (20 µM) for 30 min reversed gene expression of p53 and its down-stream p21, GADD45α and PUMA, and therefore reduced cell cycle arrest and increased cell viability.

Any chemical that induces ROS production or depletes glutathione has the potential to induce ER stress and UPR [7], and there is growing evidence that 6-OHDA can cause ER stress in various cell types [4], [8], [16], [17], [19], [38]. In addition to ROS, arylating quinones induce ER stress by activating the PERK signaling pathway, including elF2α, ATF4, and CHOP [68]. We found that 6-OHDA treatment alone activated one of the three canonical pathways of UPR, namely eIF2α-ATF4, suggesting that ER stress might be predominantly induced by Michael adduct formation by *p*-quinone. Stress conditions, such as ER stress, oxidative stress, amino acid deprivation and glucose starvation, induces both transcription and translation of ATF4 [69], [70]. Consistent with a previous report [50], we found that ATF4 was upregulated by 6-OHDA, both translationally and transcriptionally, in PC12 cells. Addition of luteolin significantly attenuated ATF4 expression at both stages.

Under ER stress, cells activate GRP78 (also known as BiP), which protects them from lethal conditions, and CHOP (also known as GADD153), which plays major roles in ER stress-induced apoptosis [71]. We observed that 6-OHDA induced the expression of GRP78 and CHOP in PC12 cells. Because UPR-mediated cell survival or death is regulated by the balance of GRP78 and CHOP expression, the preferential induction of CHOP rather than GRP78 in PC12 cells exposed to 6-OHDA indicates the possible involvement of ER stress in its cytotoxicity. Furthermore, in parallel to the protective effects, luteolin (20 µM) attenuated 6-OHDA-mediated expression of CHOP more effectively than GRP78.

The Nrf2-ARE transcriptional pathway plays an important role in the regulation of genes that control the expression of proteins critical in the detoxication and elimination of ROS and electrophiles. Quinone electrophiles are indirect inhibitors of the Keap1-Nrf2 interaction, and are believed to form covalent adducts with the sulfhydryl groups of cysteines in Keap1 by oxidation or alkylation [21]. Furthermore, direct covalent modification of Nrf2 by phosphorylation/dephosphorylation and acetylation/deacetylation affects nuclear translocation/export, and transcription activation, and degradation of Nrf2 has been reported in response to oxidative stress and toxicity [72]. Upon ER stress, PERK phosphorylates Nrf2, resulting in dissociation of the Nrf2-Keap1 complex, nuclear localization of Nrf2 and activation of transcription by Nrf2 through the antioxidant response element (ARE) [53]–[55]. In this study, we found that 6-OHDA induces modest increases in mRNA expression of Nrf2 and GCLC, and dramatic rise in HO-1 expression. Nrf2 siRNA partially decreased HO-1 expression, indicating Nrf2 and other transcription factors might be involved in this process. A growing body of evidence shows the hormetic actions of Nrf2 and HO-1. Although Nrf2 activation protects against acute toxicity and prevents or attenuates several disease states, constitutive activation leads to poor clinical outcomes [73], [74]. It has been reported that Nrf2 activation and subsequent induction of HO-1 mediate the cellular adaptive survival response to 6-OHDA-induced cell death [75]. On the other hand, sustained HO-1 over-expression contributes to the iron sequestration, intracellular oxidative stress and mitochondrial damage documented in aging-related neurodegenerative disorders, such as Alzheimer’s disease and PD [57]. Therefore, 6-OHDA-induced expression and possible activation of Nrf2 may be harmful rather than protective for PC12 cells in this study. We previously reported that luteolin itself is a mild ARE inducer, induces moderate HO-1 expression in PC12 cells, and exerts cytoprotective effects [42]. However, in the current work we found that addition of luteolin and tiron inhibited 6-OHDA-mediated HO-1 and GCLC mRNA expression, and this might be associated with their detoxifying effects. This indicates that 6-OHDA-mediated ROS production is involved in Nrf2-mediated gene expression, and that the preconditioning effects induced by luteolin mediate an adaptive response to 6-OHDA-induced cytotoxicity.

The pro-apoptotic activity of BH3-only proteins can be regulated by a variety of transcriptional and posttranslational control mechanisms. BIM, a pro-apoptotic BH3-only member of the Bcl-2 family, is required for initiation of apoptosis induced by ER stress [60]. TRB3 expression is upregulated in a variety of cell types under various stress conditions, including ER stress, nutrient deprivation, hypoxia and oxidative stress and is a critical molecule in apoptosis [61], [76]–[79]. We found that 6-OHDA stimulated the mRNA expression of BIM and TRB3, and both of these can be completely blocked by luteolin (20 µM).

The growth arrest and DNA damage-inducible protein, GADD34, forms a complex with the protein phosphatase 1 (PP1) to dephosphorylate eIF2α, and promotes protein translation in mammalian cells [62]. Consistent with a previous report that PC12 cells treated with 6-OHDA caused an increase in the transcription of GADD34 [4], addition of luteolin (20 µM) significantly decreased its induction, but did not completely prevent it.

**Figure 8:**
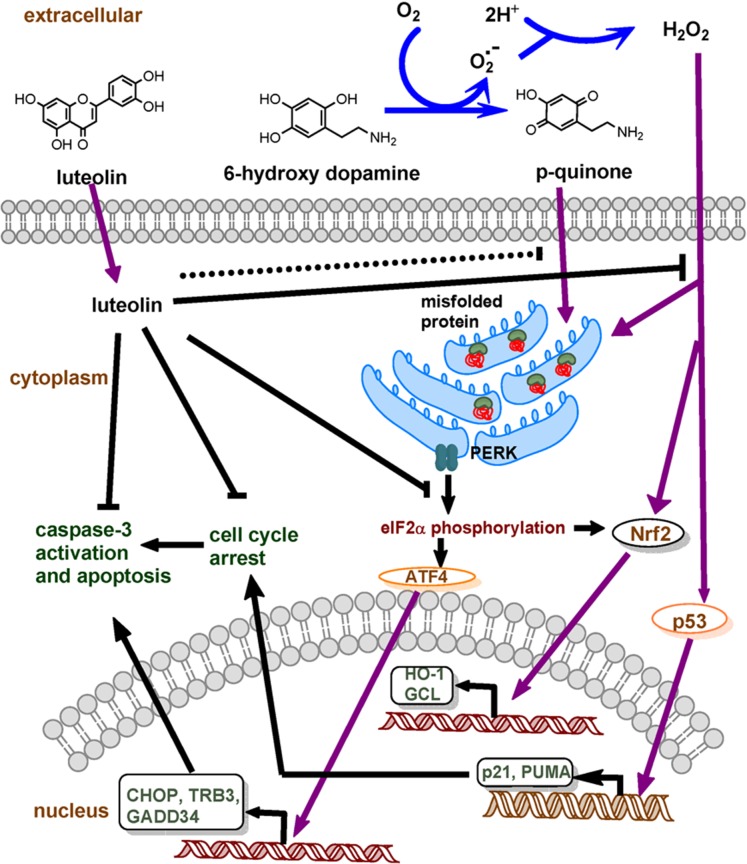
Hypothetic mechanism of luteolin in preventing 6-OHDA-induced apoptosis in PC12 cells. 6-OHDA is rapidly oxidized to generate H_2_O_2_ and *p*-quinone. Both agents activate stress response pathways, p53, Nrf2-ARE and eIF2α-ATF4-CHOP, in PC12 cells. Luteolin can directly scavenge intracellular ROS over-production and decreases the transcription of genes involved in these three pathways. The activation of caspase-3 and cell cycle arrest are subsequently attenuated and cell viability is restored.

In conclusion, we found that 6-OHDA induces transcription of genes involved in the p53, Nrf2-ARE and eIF2α-ATF4-CHOP pathways, and these subsequently cause pro-apoptotic gene over-expression and caspase-3 activation. Co-treatment of cells with luteolin (20 µM) scavenges 6-OHDA-mediated ROS production, partially attenuates ROS-dependent stress response gene expression and completely ameliorates 6-OHDA-caused cytotoxicity (**Fig. 8**).

## Supporting Information

File S1 **Supporting figures. Figure S1.** Chemical structure of luteolin. **Figure S2.** 6-OHDA causes a dose-dependent cytotoxicity in PC12 cells. PC12 cells (1×10^6^ cells/ml) were treated with 100 µM 6-OHDA in serum-free medium for 8 and 12 h at 37°C. Cell viability was measured by MTT. *p*<0.01 represents significant differences compared with vehicle control (without 6-OHDA). **Figure S3.** Effects of 6-OHDA on the transcription of p53 pathway genes. PC12 cells were cultured in serum-free medium and then incubated with 6-OHDA (100 µM) for 8 and 12 h. Levels of p53, p21, GADD45α and PUMA mRNA were measured by RT-Q-PCR and normalized to β-actin as described in Materials and Methods. *p*<0.01 represents significant differences compared with vehicle control (without 6-OHDA). **Figure S4.** Effects of luteolin on 6-OHDA-mediated protein expression of GRP78 and HO-1. (A) PC12 cells were incubated with 6-OHDA (100 µM) for 0, 2, 4, 6, 8 or 12 h. Cell lysates were prepared and immunoblotting was then carried out with antibodies against anti-GRP78, anti-HO-1 and anti-α-tubulin. (B) Cell lysates prepared from PC12 cells with indicated treatment for 12 h were subjected to GRP78, HO-1 and α-tubulin analysis as described in Materials and Methods_._ These blots are representative from one of three independent experiments. **Figure S5.** Effect of luteolin on 6-OHDA-mediated GCLC expression. (A) PC12 cells were cultured in serum-free medium and then incubated with 6-OHDA (100 µM) for 8 and 12 h. GCLC mRNA expression was measured by RT-Q-PCR and normalized to β-actin as described in Materials and Methods. (B) PC12 cells were treated with luteolin (10 or 20 µM) for 30 min before 6-OHDA (100 µM) insult for 8 h. RNA was then prepared for RT-Q-PCR analysis of GCLC. Data represent the mean ± SD of three independent experiments. *, *p*<0.05; **, *p*<0.01 represent significant differences compared with vehicle control (without 6-OHDA). #, *p*<0.05 represents significant differences compared with 6-OHDA-treated vehicle.
(DOCX)
