# Supplementary figures and images for: Luteolin Modulates 6-Hydroxydopamine-Induced Transcriptional Changes of Stress Response Pathways in PC12 Cells
Source: PLoS One. 2014 May 20;9(5):e97880. doi: 10.1371/journal.pone.0097880 (PMC4028259; doi:10.1371/journal.pone.0097880)

**Supplemental Figures**

**Figure S1**.

**Figure S2**.

| 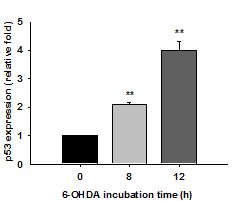 | 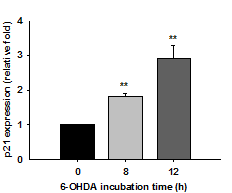 |
| --- | --- |
| 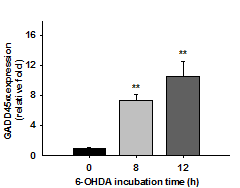 | 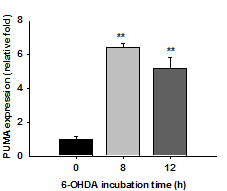 |

**Figure S3**.

A

B

**Figure S4**.

A

B

**Figure S5**.

Supplement: File S1 — Supporting figures. Figure S1. Chemical structure of luteolin. Figure S2. 6-OHDA causes a dose-dependent cytotoxicity in PC12 cells. PC12 cells (1×10^6^ cells/ml) were treated with 100 µM 6-OHDA in serum-free medium for 8 and 12 h at 37°C. Cell viability was measured by MTT. p<0.01 represents significant differences compared with vehicle control (without 6-OHDA). Figure S3. Effects of 6-OHDA on the transcription of p53 pathway genes. PC12 cells were cultured in serum-free medium and then incubated with 6-OHDA (100 µM) for 8 and 12 h. Levels of p53, p21, GADD45α and PUMA mRNA were measured by RT-Q-PCR and normalized to β-actin as described in Materials and Methods. p<0.01 represents significant differences compared with vehicle control (without 6-OHDA). Figure S4. Effects of luteolin on 6-OHDA-mediated protein expression of GRP78 and HO-1. (A) PC12 cells were incubated with 6-OHDA (100 µM) for 0, 2, 4, 6, 8 or 12 h. Cell lysates were prepared and immunoblotting was then carried out with antibodies against anti-GRP78, anti-HO-1 and anti-α-tubulin. (B) Cell lysates prepared from PC12 cells with indicated treatment for 12 h were subjected to GRP78, HO-1 and α-tubulin analysis as described in Materials and Methods. These blots are representative from one of three independent experiments. Figure S5. Effect of luteolin on 6-OHDA-mediated GCLC expression. (A) PC12 cells were cultured in serum-free medium and then incubated with 6-OHDA (100 µM) for 8 and 12 h. GCLC mRNA expression was measured by RT-Q-PCR and normalized to β-actin as described in Materials and Methods. (B) PC12 cells were treated with luteolin (10 or 20 µM) for 30 min before 6-OHDA (100 µM) insult for 8 h. RNA was then prepared for RT-Q-PCR analysis of GCLC. Data represent the mean ± SD of three independent experiments. *, p<0.05; **, p<0.01 represent significant differences compared with vehicle control (without 6-OHDA). #, p<0.05 represents significant differences compared with 6-OHDA-treated vehicle. (DOCX) [file pone.0097880.s001.docx]
